# Integrated Bioinformatics Analysis Identifies Heat Shock Factor 2 as a Prognostic Biomarker Associated With Immune Cell Infiltration in Hepatocellular Carcinoma

**DOI:** 10.3389/fgene.2021.668516

**Published:** 2021-11-30

**Authors:** Yumei Fan, Jiajie Hou, Xiaopeng Liu, Bihui Han, Yanxiu Meng, Bing Liu, Fei Chen, Yanan Shang, Pengxiu Cao, Ke Tan

**Affiliations:** ^1^ Ministry of Education Key Laboratory of Molecular and Cellular Biology, Key Laboratory of Animal Physiology, Biochemistry and Molecular Biology of Hebei Province, College of Life Sciences, Hebei Normal University, Shijiazhuang, China; ^2^ Department of Neurosurgery, The Second Hospital of Hebei Medical University, Shijiazhuang, China

**Keywords:** HSF2, hepatocellular carcinoma, prognostic biomarker, immune infiltration, immunotherapy

## Abstract

Hepatocellular carcinoma (HCC) is one of the most common malignancies and ranks as the second leading cause of cancer-related mortality worldwide. Heat shock factor 2 (HSF2) is a transcription factor that plays a critical role in development, particularly corticogenesis and spermatogenesis. However, studies examining the expression and prognostic value of HSF2 and its association with tumor-infiltrating immune cells in HCC are still rare. In the present study, we found that HSF2 expression was significantly upregulated in HCC tissues compared with normal liver tissues using the TCGA, ICGC, GEO, UALCAN, HCCDB and HPA databases. High HSF2 expression was associated with shorter survival of patients with HCC. Cox regression analyses and nomogram were used to evaluate the association of HSF2 expression with the prognosis of patients with HCC. Gene Ontology (GO) analysis, Kyoto Encyclopedia of Genes and Genomes (KEGG) analysis and gene set enrichment analysis (GSEA) revealed that HSF2 was associated with various signaling pathways, including the immune response. Notably, HSF2 expression was significantly correlated with the infiltration levels of different immune cells using the TIMER database and CIBERSORT algorithm. HSF2 expression also displayed a significant correlation with multiple immune marker sets in HCC tissues. Knockdown of HSF2 significantly inhibited the proliferation, migration, invasion and colony formation ability of HCC cells. In summary, we explored the clinical significance of HSF2 and provided a therapeutic basis for the early diagnosis, prognostic judgment, and immunotherapy of HCC.

## Introduction

Liver cancer is one of the leading causes of cancer-related mortality worldwide, with approximately 840,000 new liver cancer cases and 780,000 deaths predicted in 2018 according to the Global Cancer Statistics 2018 report (Bray et al., 2018; Forner et al., 2018). Hepatocellular carcinoma (HCC) is the most common type of primary hepatic tumor. Although the diagnosis, treatment and 5-years survival rate of patients with HCC have substantially improved in recent decades, the lack of diagnostic markers for early detection prevents the use of curative therapies, including surgical resection and liver transplantation, chemotherapy and immunotherapy (Bray et al., 2018; Fu and Wang, 2018). Currently, molecular targets are being exploited to develop novel therapies for patients with HCC, and these therapies may exert favorable curative effects and significantly prolong the patient’s survival time. Thus, the identification of novel new biological markers for the detection of early HCC, the mechanism of HCC development and progression and new molecular targets for HCC treatment are essential (Xu et al., 2021).

Cancer cells encounter a variety of internal and external stresses that normal cells do not commonly encounter (Hanahan and Weinberg, 2011). These stresses include an imbalance of protein homeostasis as a result of gene mutation, chromosomal rearrangement, oxidative stress induced by abnormal cell proliferation, protein misfolding, hypoxia caused by improper angiogenesis, and impaired degradation of proteins (Akerfelt et al., 2010; Fujimoto and Nakai, 2010). In response to multiple stimuli, heat shock factor (HSF), the principal cellular safeguard, mediates the dynamic expression of various molecular chaperones, also known as heat shock proteins (HSPs), which are responsible for exerting subsequent downstream effects, including stress-related cytoprotective events, the folding and assembly of nascent polypeptides and the intracellular transport of proteins (Gomez-Pastor et al., 2018; Puustinen and Sistonen, 2020). HSF1 plays an important role in the initiation, promotion and progression of different types of cancer (Puustinen and Sistonen, 2020; Wang et al., 2020). HSF1 knockdown significantly reduces tumor growth and prolongs survival when cells are exposed to various carcinogens (Dai, 2018; Joutsen and Sistonen, 2019; Wang et al., 2020). Therefore, HSF1 has been recognized as a potential therapeutic target for antitumor therapy. A plethora of screening studies have identified many molecules that act as inhibitors of HSF1 (Kijima et al., 2019). In contrast, HSF2 has been shown to play key roles in regulating the ubiquitin proteasome pathway and differentiation (Mathew et al., 1998). HSF2 is also associated with embryogenesis and spermatogenesis (Rallu et al., 1997; Widlak and Vydra, 2017). HSF2-null mice display defects in spermatogenesis (Sarge et al., 1994; Wang et al., 2003; Bjork et al., 2010). Increased apoptosis of spermatocytes and defects in the maturation of male germ cells were observed in HSF2-null mice (Sarge et al., 1994; Wang et al., 2003; Bjork et al., 2010). In the testis, HSF2 mediates the expression of several HSPs and Y chromosomal multicopy genes (SLX, SLY and SSTY2) that are important for spermatogenesis (Akerfelt et al., 2008). HSF2-null mice also present brain abnormalities characterized by enlarged ventricles, a small hippocampus, and mispositioning of neurons (Kallio et al., 2002; Chang et al., 2006). Recent studies implicate a more extensive role for HSF2 and suggest that HSF2 forms heterotrimers with HSF1 to induce the expression of HSPs or other genes (Sistonen et al., 1994; Ostling et al., 2007; Sandqvist et al., 2009). The functional cooperation of HSF1 and HSF2 and their coinvolvement in regulating proteostasis, together with the identification of HSF1 as a promising and effective antitumor drug, indicate that HSF2 may play a role in tumorigenesis. However, compared with HSF1, HSF2 has not been extensively investigated in cancer, and its function and molecular mechanisms in oncogenesis are largely unknown.

A few studies have shown altered expression of HSF2 in cancer. Upregulated expression of HSF2 was observed in lung cancer, esophageal squamous cell carcinoma (ESCC) and gliomas, whereas downregulated expression of HSF2 was detected in prostate cancer (Mustafa et al., 2010; Björk et al., 2016; Zhong et al., 2016; Meng et al., 2017). Because HSF2 silencing changed the stability of p53 and its cooperation with HSF1, HSF2 likely affects tumorigenesis (Lecomte et al., 2010). Hence, a comprehensive analysis of HSF2 expression and the prognostic value of HSF2 in different types of cancer is urgently needed. The present study identified the expression of HSF2 mRNA in tumor tissues and compared it to normal liver tissues. Increased HSF2 expression was correlated with various clinicopathological parameters of HCC. Kaplan-Meier analyses suggested that HSF2 expression is an independent predictor of the survival rate of patients with HCC. Additionally, receiver operating characteristic (ROC) curve analyses of HSF2 indicated that HSF2 may be a diagnostic indicator of HCC. Moreover, Gene Ontology (GO), Kyoto Encyclopedia of Genes and Genomes (KEGG) and gene set enrichment analysis (GSEA) revealed that HSF2 was involved in various signaling pathways and the immune response. In addition, HSF2 expression was positively correlated with the infiltration of different immune cells. Taken together, the upregulation of HSF2 expression is significantly associated with the progression and poor prognosis of HCC and immune cell infiltration, indicating that HSF2 may represent a target for early diagnosis, prognostic predictions and immunotherapy.

## Materials and Methods

### Data Collection

Genomic data from The Cancer Genome Atlas (TCGA) were downloaded and analyzed using the NCI Genomic Data Commons (https://portal.gdc.cancer.gov). The corresponding clinical data were retrieved using the University of Santa Cruz (UCSC) Xena platform. We also searched HCC gene expression data from the Gene Expression Omnibus (GEO) database (http://www.ncbi.nlm.nih.gov/geo/), including the GSE6764, GSE19665, GSE112790 and GSE121248 datasets. The box plots are implemented using the R software package ggplot2. For the ICGC-LIRI-JP cohort, RNA-seq and clinical data were downloaded from the International Cancer Genome Consortium (ICGC) portal. All data were downloaded from publicly accessible databases, and no ethics committee approval was required.

### UALCAN Database

UALCAN (http://ualcan.path.uab.edu/) is a publicly accessible and interactive web portal for analyzing transcriptome data from various types of cancer (Liu B. et al., 2021; Fan et al., 2021). HSF2 mRNA levels in HCC and normal liver tissues and different clinicopathological parameters were explored using UALCAN.

### HCCDB Database

The HCCDB database (http://lifeome.net/database/hccdb/home.html), which contains fifteen public HCC transcriptional expression datasets, is a gene expression atlas of HCC that enables visualization of the findings from multiple bioinformatics analyses. We used the HCCDB database to explore the expression patterns and prognostic value of HSF2 in HCC.

### Gene Expression Profiling Interactive Analysis

GEPIA2 (http://gepia2.cancer-pku.cn), an online cancer microarray database retrieved from the UCSC Xena server, was used to analyze the relationship between HSF2 and other genes.

### cBioPortal Database

CBioPortal (www.cbioportal.org) is an online open access database that contains both sequencing and pathological data on 30 different cancers and allows researchers to interactively analyze multidimensional cancer genomics data. According to the online instructions provided by cBioPortal, the genomic profiles, including the genetic alterations, survival curves and correlations of HSF2, were investigated.

### Kaplan-Meier Plotter Analysis

The prognostic value of HSF2 mRNA expression was investigated using the Kaplan-Meier plotter database (www.kmplot.com), which contains gene expression data and survival information for clinical patients with cancer. Patients with liver cancer were divided into two groups (high and low expression) according to the median HSF2 expression level. The OS, recurrence-free survival (RFS), progression-free survival (PFS) and disease-specific survival (DSS) of patients with liver cancer were analyzed by constructing a Kaplan-Meier survival plot and calculating hazard ratios (HRs), 95% confidence intervals and log-rank *p*-values. The relationship between HSF2 expression and clinicopathological parameters, i.e., sex and clinical stage of HCC, was also examined using the Kaplan-Meier plotter database. *p* < 0.05 was considered to indicate a statistically significant result.

### Cox Regression Analysis and Construction and Evaluation of a Nomogram

The association between HSF2 expression and the OS of patients with HCC was evaluated by performing univariate and multivariate Cox regression analyses. A forest plot was generated to show the *p* value, HR and 95% CI of each clinicopathological parameter using the “forestplot” R package. According to the clinical characteristics, such as age, sex, T stage, N stage and M stage, we generated a nomogram to predict the probability of 1-, 3-, and 5-years OS using the R package “rms”(https://www.rdocumentation.org/packages/rms). The concordance index (C-index) was calculated to assess the predictive accuracy.

### Tumor Immune Estimation Resource

The correlations between HSF2 expression and the infiltration of each type of immune cell, including B cells, CD4^+^ T cells, CD8^+^ T cells, neutrophils, macrophages and dendritic cells, were investigated using the TIMER web-based platform (https://cistrome.shinyapps.io/timer/).

### Immune Cell Infiltration Assessment With the CIBERSORT Algorithm

The CIBERSORT tool (https://cibersort.stanford.edu/), which contains 547 genes and 22 human immune cell subpopulations, was applied to characterize the immune cell composition based on a validated leukocyte gene signature matrix. Our current analysis examined the proportions of tumor-infiltrating immune cells in HCC using the CIBERSORT algorithm and investigated the correlations between HSF2 expression and immune cell subpopulations. A *p*-value < 0.05 was set as the criterion to select lymphocytes possibly affected by HSF2 expression.

### Immunohistochemistry Analysis

The HSF2 protein levels in normal liver and HCC tissues from the Human Protein Atlas (HPA) database (https://www.proteinatlas.org/) were analyzed based on IHC staining data.

### Gene Ontology, Kyoto Encyclopedia of Genes and Genomes and Gene Set Enrichment Analysis

GO analysis, KEGG analysis and GSEA were applied to investigate the biological functions and potential mechanisms of HSF2 in HCC. GO analysis is a powerful bioinformatics method that annotates and categorizes genes according to biological processes (BPs), cellular components (CCs) and molecular functions (MFs) (Chen et al., 2021). GSEA was used to investigate the potential mechanisms of hepcidin. The GO, KEGG and GSEA results were explored using the R package ClusterProfiler (Chen et al., 2021).

### GeneMANIA Analysis

GeneMANIA (http://www.genem ania.org), a flexible web interface, generates a list of genes with similar functions to the query gene and constructs a functional association network. In the present study, a gene-gene interaction network for HSF2 was constructed to evaluate the functions of related genes using GeneMANIA.

### Cell-Based Experiments

HCC cell lines, including HepG2 and Hep3B cells, were obtained from the American Type Culture Collection (ATCC, Manassas, VA, United States). The cells were cultured in Dulbecco’s Modified Eagle’s Medium (DMEM, Gibco, United States) supplemented with 10% fetal bovine serum (FBS, ExCell Bio, Shanghai, China) at 37°C with 5% CO_2_ (Tan et al., 2015). HepG2 and Hep3B cells were infected with a lentivirus expressing HSF2-shRNA for 72 h and screened with puromycin (Sigma, St. Louis, MO, United States). Puromycin-resistant clones were amplified and screened through western blotting with an anti-HSF2 antibody (1:500, Cat# K003476P, Solarbio, China). The lentivirus was generated by Genechem Co., Ltd. (Shanghai, China).

A CCK-8 kit (MCE, Shanghai, China) was used to examine cell viability according to the manufacturer’s instructions. HepG2 and Hep3B cells were seeded on 96-well plates containing 100 μl of DMEM at a density of 6 × 10^3^ cells/well. Colony formation assays were performed as previously described (Liu M. et al., 2021). HepG2 and Hep3B cells (8 × 10^2^ cells/well) were seeded into 6-well plates. For the wound healing assay, monolayers of HepG2 and Hep3B cells (5 × 10^5^ cells/well) with or without HSF2 knockdown were scratched using a pipette tip. After 24 h, representative images of HepG2 and Hep3B cells were captured using a microscope. Cell invasion was examined using 24-well Transwell units with polycarbonate filters (pore size, 8.0 μm; Corning, NY, United States) as described in a previous study (Wang et al., 2017). Briefly, the upper Transwell inserts were coated with 100 μl of Matrigel basement membrane (BD Biosciences, San Diego, CA, United States), and then 100 μl of serum-free DMEM medium was added. HepG2 and Hep3B cells (1× 10^5^) were seeded on the upper Transwell inserts. DMEM (600 μl) containing 5% FBS as a chemoattractant was added to the lower chamber. HepG2 and Hep3B cells were allowed to invade from the upper chamber for 24 h; then, 4% paraformaldehyde was used to fix the cells on the filters, and a 0.05% crystal violet solution was used to stain the cells. Invading cells were counted using a microscope.

### Statistical Analysis

The survival results obtained from Kaplan-Meier plotter, HCCDB and GEPIA are displayed as the HR and P or Cox *p*-values calculated using a log-rank test. Pearson’s correlation coefficient, Spearman’s correction coefficient, Kruskal-Wallis test, Wilcox test and statistical significance were used to evaluate the correlation of gene expression, and the strength of the correlation was determined using the absolute values. Potential immune checkpoint blockade (ICB) response was predicted with the TIDE (Tumor Immune Dysfunction and Exclusion) algorithm using R software ggplot2 (v3.3.3) and ggpubr (0.4.0). The results were considered statistically significant at *p < 0.05, **p < 0.01, and ***p < 0.001.

## Results

### Heat Shock Factor 2 Expression Levels in Hepatocellular Carcinoma

**FIGURE 1:**
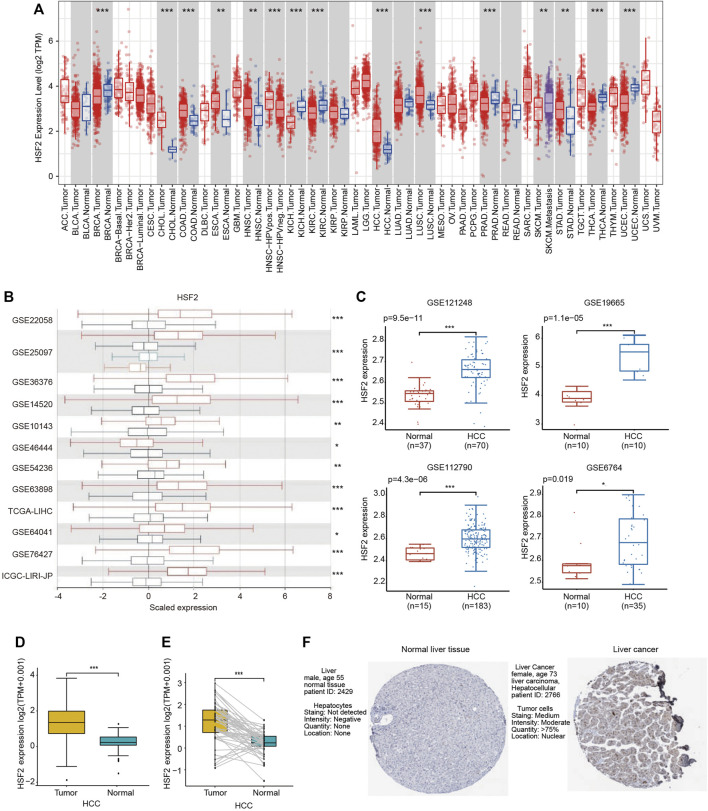
HSF2 expression in HCC. **(A)** Increased or decreased expression of HSF2 in different types of tumors compared to normal tissues in the TIMER database. **(B)** Expression of HSF2 in HCC and normal tissues in the HCCDB database. **(C)** HSF2 expression in HCC (GSE121248, *n* = 70; GSE19665, *n* = 10; GSE112790, *n* = 183; GSE6764, *n* = 35) and normal tissues (GSE121248, *n* = 37; GSE19665, *n* = 10; GSE112790, *n* = 15; GSE6764, *n* = 10) was investigated by analyzing four GEO datasets. **(D)** Analysis of HSF2 expression in HCC (*n* = 371) and adjacent normal liver tissues (*n* = 50) in TCGA database. **(E)** HSF2 expression in 50 pairs of HCC and adjacent normal tissues from TCGA database (*n* = 50). **(F)** HSF2 expression in HCC and normal liver tissues from the HPA database. *p < 0.05, **p < 0.01, ***p < 0.01.

Gene expression was first analyzed in the TIMER database to validate the differential expression of HSF2 between various tumor tissues and adjacent normal tissues (Figure 1A). HSF2 expression was significantly upregulated in cholangiocarcinoma (CHOL), colon adenocarcinoma (COAD), esophageal carcinoma (ESCA), head and neck squamous cell carcinoma (HNSC), HCC, lung squamous cell carcinoma (LUSC) and stomach adenocarcinoma (STAD) and downregulated in breast cancer (BRCA), kidney chromophobe renal cell carcinoma (KICH), kidney renal clear cell carcinoma (KIRC), prostate adenocarcinoma (PRAD), thyroid carcinoma (THCA) and uterine corpus endometrial carcinoma (UCEC) (Figure 1A). We further validated the differential expression of HSF2 in the HCCDB database. HSF2 was markedly overexpressed in HCC samples compared with normal liver samples in 12 different datasets (Figure 1B). Consistently, higher expression of HSF2 was observed in HCC tissues than in normal tissues in other GEO datasets, including GSE6764, GSE19665, GSE112790, and GSE121248 (Figure 1C). A further evaluation of HSF2 expression in HCC was performed using the data directly obtained from TCGA database, and HSF2 expression was substantially increased in HCC tissues (Figure 1D). Moreover, marked upregulation of HSF2 expression was detected in 50 paired tumor samples and adjacent normal samples (Figure 1E). We analyzed IHC images from the HPA database to further explore the expression of HSF2 protein in HCC. HSF2 protein expression was significantly upregulated in liver cancer tissues compared with normal liver tissues (Figure 1F). In summary, the aforementioned results indicated that HSF2 was expressed at higher levels in HCC than in healthy controls.

### Association of Heat Shock Factor 2 Expression With the Clinicopathological Parameters of Patients With Hepatocellular Carcinoma

**FIGURE 2:**
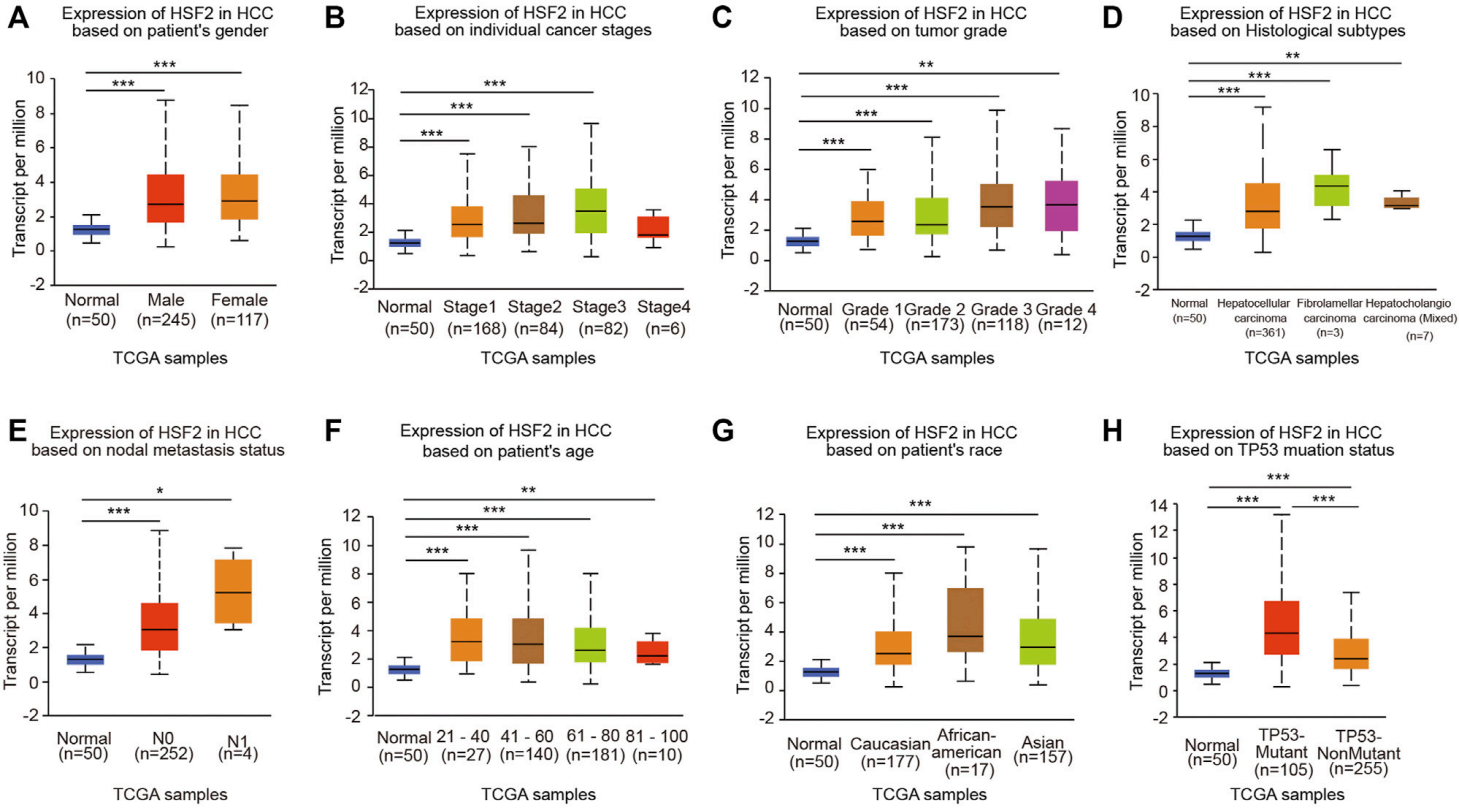
HSF2 expression among different groups of patients stratified based on clinical parameters. The analyses of HSF2 expression based on **(A)** sex (female, *n* = 117; male, *n* = 245), **(B)** cancer stage (normal individuals, *n* = 50; stage 1, *n* = 168; stage 2, *n* = 84; stage 3, *n* = 82; stage 4, *n* = 6), **(C)** tumor grade (normal individuals, *n* = 50; grade 1, *n* = 54; grade 2, *n* = 173; grade 3, *n* = 118; grade 4, *n* = 14), **(D)** histological subtype (normal individuals, *n* = 50; hepatocellular carcinoma, *n* = 361; fibrolamellar carcinoma, *n* = 3; hepatocholangiocarcinoma, *n* = 7), **(E)** metastasis (normal individuals, *n* = 50; N0, *n* = 252; N1, *n* = 4), **(F)** age (normal individuals, *n* = 50; 21–40 years, *n* = 27; 41–60 years, *n* = 140; 61–80 years, *n* = 181; and 81–100 years, *n* = 10), **(G)** race (normal individuals, *n* = 50; Caucasian, *n* = 177; African-American, *n* = 17; Asian, *n* = 157), and **(H)** TP53 mutation status (normal individuals, *n* = 50; TP53-nonmutant, *n* = 255; TP53-mutant, *n* = 105) using TCGA-HCC dataset are shown. N0, no regional lymph node metastasis; N1, metastases in 1–3 axillary lymph nodes. *p < 0.05, **p < 0.01, ***p < 0.01.

We next analyzed the relationship between HSF2 expression and clinicopathological parameters using the UALCAN database. HSF2 mRNA levels were significantly increased in HCC samples from both males and females compared with the corresponding normal controls (Figure 2A). After stratification according to individual cancer stages, an increase in HSF2 expression was found in patients with stage 1, 2, 3 and 4 HCC compared to normal controls (Figure 2B). Regarding tumor grade, HSF2 expression was significantly elevated in patients with grades 1, 2, 3 and 4 HCC, and the highest levels of HSF2 were observed in patients with grade 4 HCC (Figure 2C). Patients with HCC presenting more advanced tumor grades tended to express higher levels of HSF2 than those with less advanced tumors. After stratification based on histological subtypes, HSF2 was expressed at higher levels in HCC, fibrolamellar carcinoma and hepatocholangiocarcinoma than in normal controls (Figure 2D). In terms of the nodal metastasis status, increased HSF2 expression was observed in patients with HCC classified as N0 and N1 (Figure 2E). In terms of age, HSF2 was dramatically upregulated in patients with HCC compared to normal controls in different age groups (21–40, 41–60, 61–80 and 81–100 years) (Figure 2F). In addition, upregulation of HSF2 expression was observed in patients with HCC of three races, including Caucasian, African-American, and Asian patients (Figure 2G). Moreover, a significant increase in HSF2 expression was detected in patients with TP53-mutant HCC compared to patients with TP53 wild-type HCC and normal controls (Figure 2H).

### High Heat Shock Factor 2 Expression Predicts a Poor Prognosis for Patients With Hepatocellular Carcinoma

**FIGURE 3:**
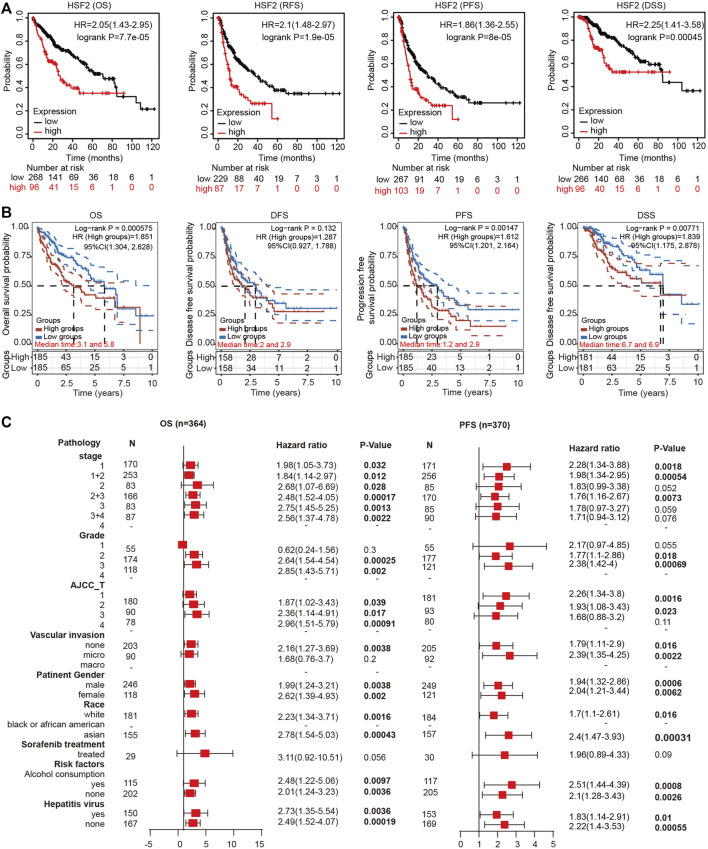
High HSF2 expression was correlated with a poor survival rate in patients with HCC. **(A)** OS, RFS, PFS and DSS of HCC cohorts, as determined using Kaplan-Meier plotter analysis. **(B)** OS, PFS, DFS and DSS of HCC cohorts in TCGA database are shown using the R software package survival and survminer. **(C)** Forest plots showing the association between HSF2 expression and clinicopathological parameters of patients with HCC.

We investigated the effects of HSF2 expression on the survival rate to explore the correlation between HSF2 expression and the prognosis of patients with HCC. The Kaplan-Meier plotter analysis revealed that patients with HCC presenting upregulated HSF2 levels experienced shorter OS, RFS, PFS and DSS than those without HSF2 upregulation (Figure 3A). Consistent with the aforementioned results, the survival analysis based on TCGA database indicated that patients with HCC presenting high HSF2 expression had a poor prognosis, including OS, PFS, and DSS (Figure 3B). Moreover, the results from the UALCAN database suggested that HSF2 overexpression was significantly correlated with a shorter OS for patients with HCC of different tumor grades and sexes (Supplementary Figure S1A). Then, we further assessed the prognostic value of HSF2 in patients with HCC using the HCCDB database. As expected, patients with HCC presenting high HSF2 expression in the TCGA-LIHC and ICGC-LIRI-JP cohorts had a poor prognosis (Supplementary Figures S1B,C). In contrast, HSF2 had no effect on OS in normal individuals in these analyses (Supplementary Figures S1B,C).

### Prognostic Value of Heat Shock Factor 2 Based on Different Clinicopathological Characteristics

Based on the differential expression of HSF2 and its significant prognostic value in HCC, we investigated the relationship between the expression of HSF2 and different clinicopathological characteristics of HCC using the Kaplan-Meier plotter database. Specifically, upregulated expression of HSF2 was significantly associated with shorter OS of patients with HCC of stages 1, 2, 1+2, 2+3, 3 and 3+4, grades 2 and 3, and AJCC-T stage 1, 2 and 3 (Figure 3C). In addition, high HSF2 expression was significantly related to a shorter PFS for patients with stages 1, 1+2 and 2+3, grades 2 and 3, and AJCC-T stage 1 and 2 HCC (Figure 3C). Moreover, high HSF2 expression was related to poor OS and PFS rates in patients, regardless of race (white and Asian), sex, alcohol abuse, and hepatitis viral infection (Figure 3C). These data suggested that high HSF2 expression may be an independent predictor of a poor prognosis for patients with HCC.

### Cox Hazard Regression Analysis and Construction of the Nomogram Model

**FIGURE 4:**
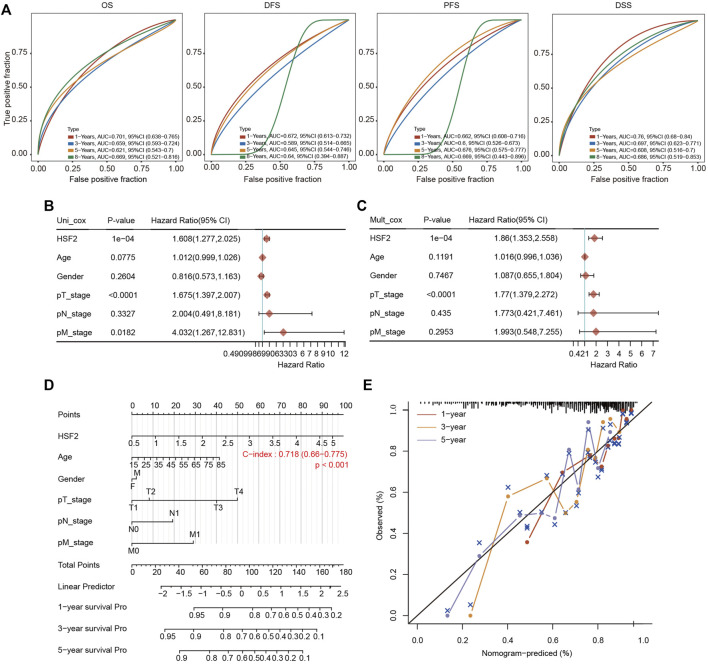
Establishment of the prognostic nomogram. **(A)** ROC curves for HSF2 mRNA expression in HCC cohorts using the R software package timeROC. **(B,C)** Univariate and multivariate Cox regression analysis of clinicopathological variables and HSF2 expression in patients with HCC. The forests are used to show the *p* value, HR and 95% CI through the R package forestplot. **(D)** Nomogram for predicting the 1-, 3-, and 5-years OS of patients with HCC using the R software package rms. **(E)** Calibration curves for the 1-, 3-, and 5-years OS of patients with HCC.

A ROC curve analysis was conducted to analyze the diagnostic accuracy of HSF2 expression for HCC diagnosis. In a comparison between tumor tissues and matched adjacent normal tissues in the TCGA cohort, the areas under the curve (AUCs) for 1-year, 3-years, 5-years and 8-years OS were 0.701, 0.659, 0.621 and 0.669, respectively (Figure 4A). The AUC values for 1-year, 3-years, 5-years and 8-years DFS were 0.672, 0.589, 0.645 and 0.64, respectively (Figure 4A). The AUC values for 1-year, 3-years, 5-years and 8-years PFS were 0.662, 0.6, 0.676 and 0.669, respectively (Figure 4A). The AUC values for 1-year, 3-years, 5-years and 8-years DSS were 0.76, 0.697, 0.608 and 0.686, respectively (Figure 4A).

We performed univariate Cox and multivariate Cox regression analyses to further evaluate whether HSF2 expression was an independent prognostic factor for patients with HCC. In the univariate Cox regression analysis, HSF2 expression, age, T stage and M stage were significantly associated with the OS of patients with HCC (Figure 4B). In the multivariate Cox regression analysis, HSF2 expression and T stage exhibited obvious correlations with the OS of patients with HCC (Figure 4C).

Based on the multivariate regression analysis, we constructed a novel nomogram model to predict the 1-, 3-, and 5-years OS rates of patients with HCC (Figure 4D). The C index of the prognostic nomogram was 0.718 (Figure 4D). The calibration plots of the nomogram showed good agreement between the actual and nomogram-predicted 1-, 3- and 5-years survival rates (Figure 4E).

### Heat Shock Factor 2 Genetic Alterations and Neighboring Gene Network in Hepatocellular Carcinoma

**FIGURE 5:**
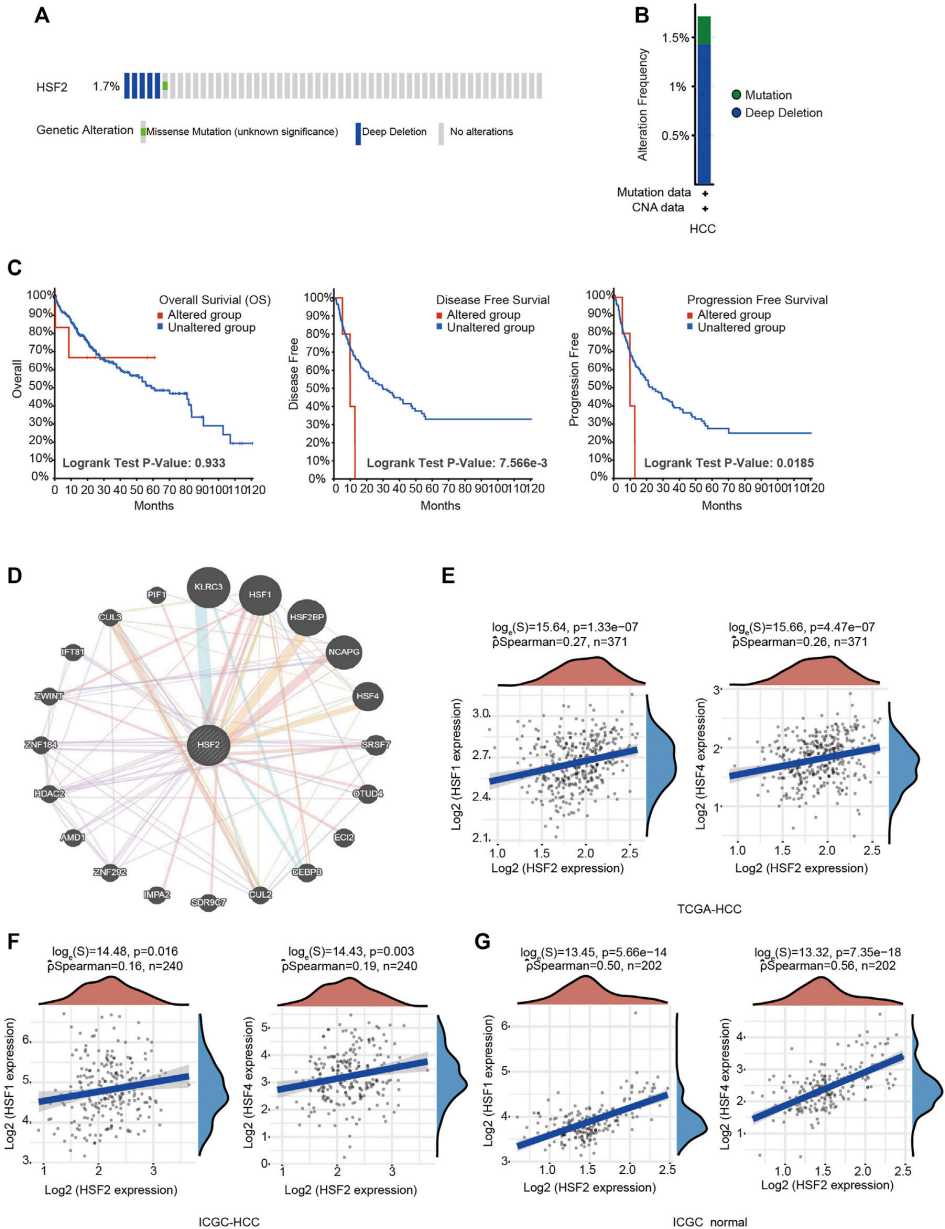
Frequency of HSF2 alterations and analysis of neighboring gene networks in HCC. **(A)** OncoPrint visual summary of alterations in a query of HSF2 from the cBioPortal database. **(B)** Summary of HSF2 genetic alterations in patients with HCC. **(C)** Kaplan-Meier plots comparing OS, DFS and PFS of patients with or without HSF2 gene alterations. **(D)** The gene-gene interaction network of HSF2 was constructed using GeneMANIA. **(E)** Scatterplots show the correlations between HSF2 expression and HSF1 and HSF4 expression in the TCGA-HCC database (*n* = 371) using the R software package ggstatsplot. **(F,G)** Scatterplots show the correlations between HSF2 expression and HSF1 and HSF4 expression in HCC (*n* = 240) and normal liver tissues (*n* = 202) in the ICGC database using the R software package ggstatsplot.

The frequency of HSF2 alterations in HCC was analyzed using cBioPortal. A total of 372 patients with HCC were analyzed (HCC, TCGA, and PanCancer Atlas). Genetic variations in HSF2 were detected at an incidence rate of 1.71%, and deep depletion was the most common type (Figures 5A,B). However, the results of the Kaplan-Meier plotter analysis indicated that although a statistically significant difference in OS was not observed between patients with HCC presenting with or without alterations in HSF2, patients with HCC presenting alterations in HSF2 experienced shorter DFS and PFS (Figure 5C). We then examined the methylation alteration of HSF2 in HCC. HCC samples showed elevated DNA methylation level of HSF2 compared with normal individuals using the UALCAN database (Supplementary Figure S2A). Next, we constructed the gene-gene interaction network for HSF2 and the altered neighboring genes using GeneMANIA. The 20 most frequently altered genes were closely correlated with HSF2 (Figure 5D). Importantly, other members of the HSF family, including HSF1 and HSF4, were shown to interact with HSF2 (Figure 5D). The correlations between HSF2 and HSF1 and HSF4 were evaluated using the TCGA database and ICGC dataset (Figures 5E–G). HSF2 expression was strongly associated with HSF1 and HSF4 expression in HCC tumors and normal liver tissues (Figures 5E–G).

### Molecular Mechanisms of Heat Shock Factor 2 in Hepatocellular Carcinoma

**FIGURE 6:**
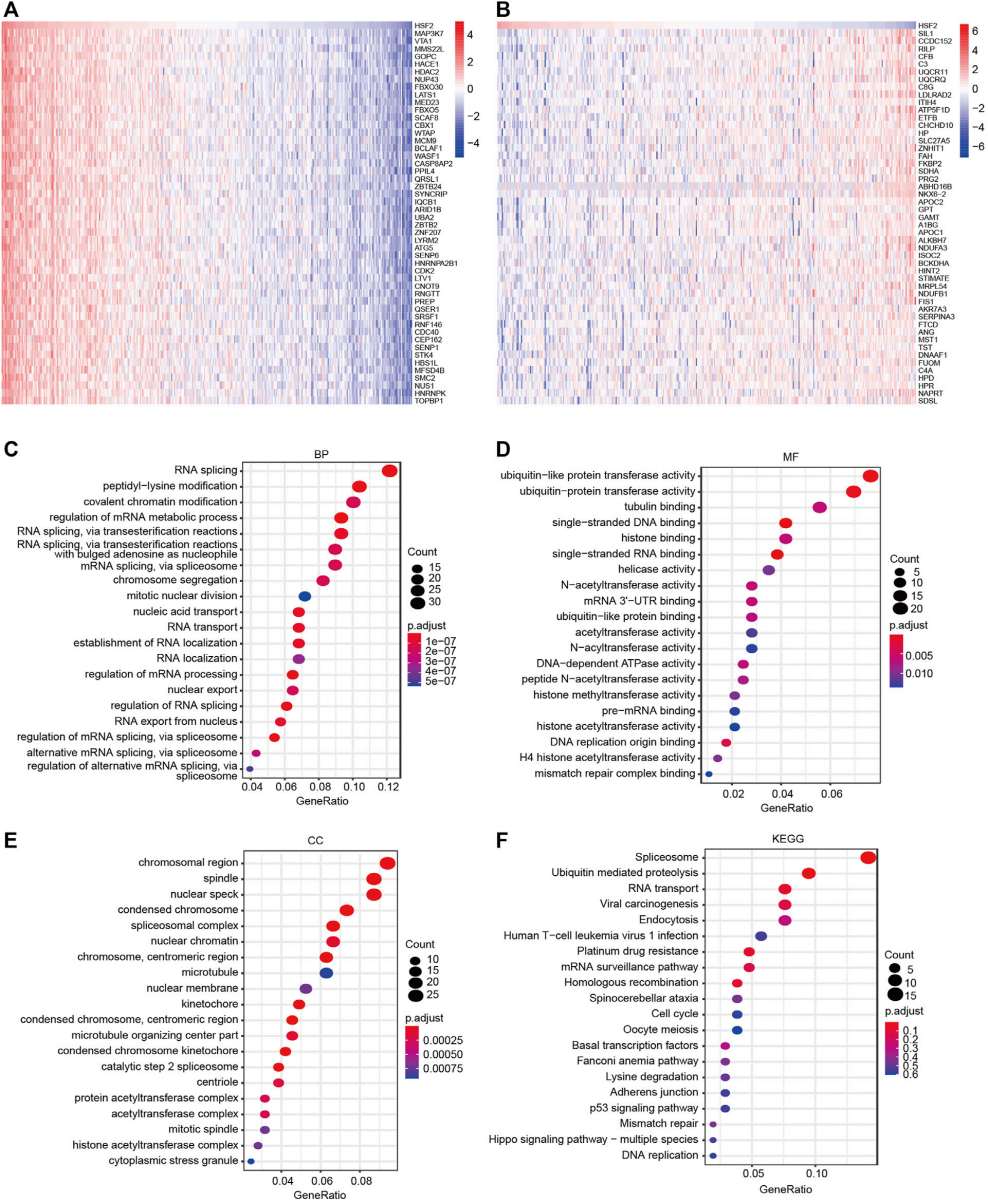
GO and KEGG enrichment analyses of HSF2. **(A–B)** Heatmaps indicating the top 50 genes positively and negatively correlated with HSF2 in HCC. **(C–E)** Top 20 enriched terms in the BP, MF and CC categories in HCC. **(F)** Top 20 pathways enriched in the KEGG analysis of HCC.

We performed GO and KEGG pathway analyses with data obtained from the TCGA dataset to understand the role and molecular mechanism of HSF2 in HCC. The top 50 genes that were positively or negatively associated with HSF2 are shown in Figures 6A and B, respectively. GO analysis is a powerful bioinformatics tool to explore the BPs, CCs and MFs of HSF2. The top 5 enriched BP terms were RNA splicing, peptidyl-lysine modification, covalent chromatin modification, regulation of mRNA metabolic process, and RNA splicing via transesterification reactions (Figure 6C). The top 5 enriched MF terms were ubiquitin-like protein transferase activity, ubiquitin-protein transferase activity, tubulin binding, single-stranded DNA binding, and histone binding (Figure 6D). The CC enrichment analysis showed that HSF2 was significantly correlated with chromosomal regions, spindles, nuclear specks, condensed chromosomes and spliceosomal complexes (Figure 6E). In addition, KEGG pathway analysis revealed that HSF2 was involved in signaling pathways related to carcinogenesis, such as ubiquitin-mediated proteolysis, viral carcinogenesis, endocytosis, platinum drug resistance, homologous recombination, cell cycle, p53 signaling pathway, mismatch repair, Hippo signaling pathway and DNA replication (Figure 6F). These findings indicate that HSF2 plays a role in tumor development and progression.

### Heat Shock Factor 2-Related Signaling Pathways Obtained From the Gene Set Enrichment Analysis

**FIGURE 7:**
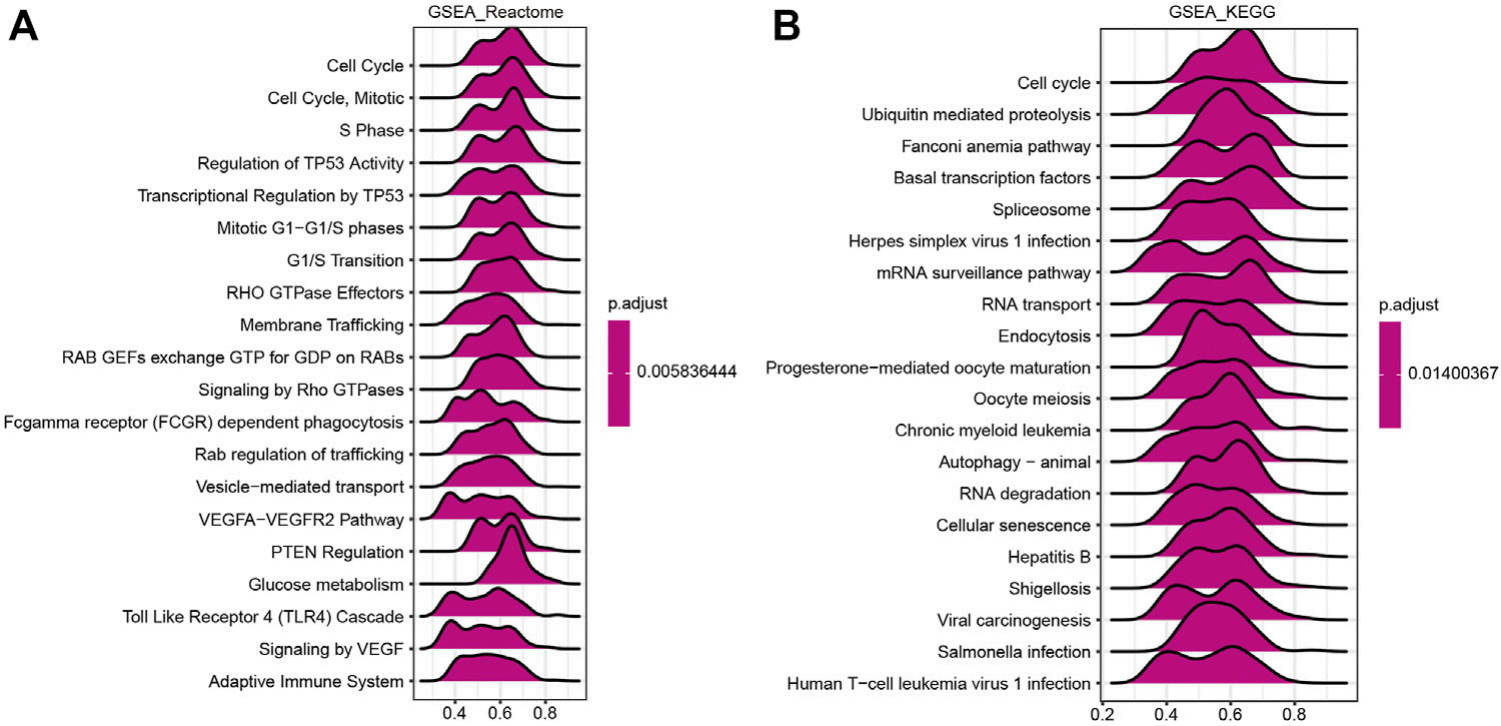
Merged enrichment plots from GSEA. **(A,B)** Merged plots showing the pathways associated with HSF2 expression based on Reactome and KEGG analyses of HCC.

A GSEA was performed to further investigate the molecular mechanisms influenced by HSF2 in HCC. GO analysis revealed that HSF2 was significantly involved in pathways that included mitotic sister chromatid segregation, RNA splicing, DNA damage checkpoints, cell cycle checkpoints, and the ubiquitin ligase complex (Supplementary Figure S2B). Similarly, among the Reactome terms, several pathways related to the immune system, including the Toll-like receptor 4 (TLR4) cascade and the adaptive immune system, were identified (Figure 7A). Moreover, KEGG enrichment analysis showed that HSF2 was related to the cell cycle, ubiquitin-mediated proteolysis, the Fanconi anemia pathway, basal transcription factors, the spliceosome, etc. In addition, herpes simplex virus 1 infection, hepatitis B, shigellosis, Salmonella infection, and human T-cell leukemia virus 1 infection, which are related to the immune response, were also correlated with HSF2 in the KEGG pathways (Figure 7B). These results indicate a potential relationship between HSF2 and immune response regulation.

### Correlation Analysis Between Heat Shock Factor 2 Expression and Infiltrating Immune Cells

**FIGURE 8:**
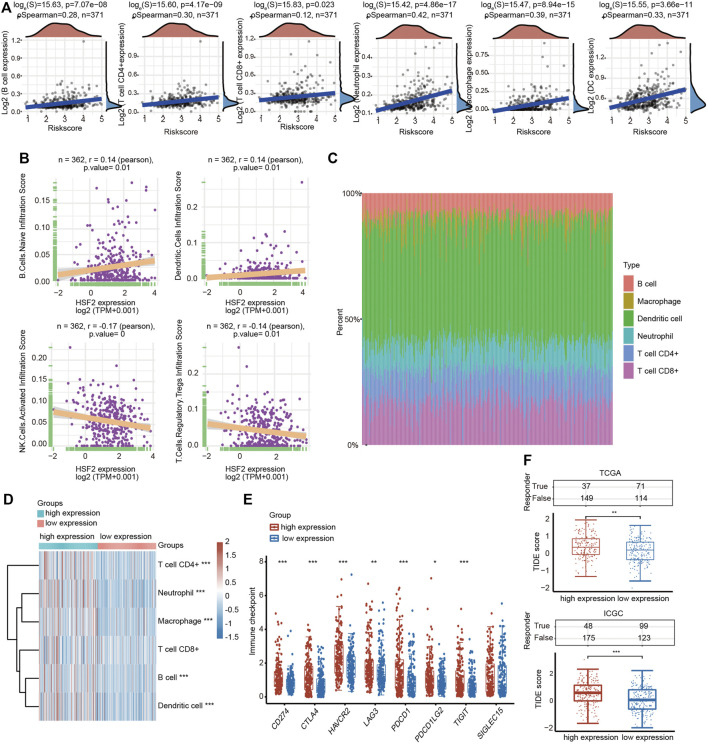
Relationship between HSF2 expression and infiltration levels of immune cells in HCC. **(A)** HSF2 expression was significantly and positively associated with the infiltration levels of B cells, CD8^+^ T cells, CD4^+^ T cells, macrophages, neutrophils and dendritic cells in HCC according to the TIMER database analysis using the R software package ggstatsplot (*n* = 371). **(B)** HSF2 expression was positively or negatively correlated with the infiltration of different immune cells in HCC according to the CIBERSORT algorithm. **(C)** Visualization of the infiltrating levels of different immune cells in the HCC samples. **(D)** Comparison of the proportions of immune cells in the HSF2 high-expression and low-expression groups. **(E)** The expression of multiple immune checkpoint genes between the HSF2 low-expression group and the high-expression group. These results were obtained using the R software (v4.0.3) package ggplot2 and pheatmap. **(F)** The TIDE score between the HSF2 low-expression group and the high-expression group based on TCGA and ICGC databases. *p < 0.05, **p < 0.01, ***p < 0.01.

A growing number of studies have proven an immunoregulatory effect of tumor-infiltrating immune cells on the development and progression of tumors (Mendillo et al., 2012). Therefore, we analyzed the correlation between HSF2 expression and six major types of infiltrating immune cells, including B cells, CD4^+^ T cells, CD8^+^ T cells, neutrophils, macrophages and dendritic cells, in patients with HCC (Figure 8A). HSF2 expression showed a significant positive correlation with the infiltration levels of B cells, CD8^+^ T cells, CD4^+^ T cells, macrophages, neutrophils, and dendritic cells in HCC (Figure 8A). We estimated the association between HSF2 and immune cell infiltration using CIBERSORT to further assess the relationship between HSF2 and the tumor microenvironment (TME). HSF2 expression was significantly and positively correlated with the infiltration levels of native B cells and dendritic cells but negatively correlated with the infiltration levels of activated NK cells and Treg cells in HCC (Figure 8B). After stratification according to HSF2 expression, patients with HCC were separated into high-expression and low-expression groups. The percentage abundances of tumor infiltrating immune cells in each sample and different types of immune cells identified using TIMER are shown in different colors (Figure 8C). Increased percentages of infiltrating B cells, CD4^+^ T cells, neutrophils, macrophages and dendritic cells were observed in the HSF2 high-expression group compared with the HSF2 low-expression group (Figure 8D). These findings indicate that HSF2 may recruit immune cells to the TME of HCC.

### Correlation Between Heat Shock Factor 2 Expression and Different Gene Markers of Immune Cell Subsets

We further explored the relationships between HSF2 expression and various tumor-infiltrating immune cells in HCC using the TIMER and GEPIA databases by analyzing the different immune cell gene markers. The correlation was adjusted for tumor purity due to its effect on the immune cell infiltration analysis. HSF2 expression was significantly associated with the expression of most gene markers of T cells, CD8^+^ T cells, B cells, monocytes, tumor-associated macrophages (TAMs), M1 and M2 macrophages, neutrophils, NK cells and dendritic cells in patients with HCC after adjusting for tumor purity (Table 1). Consistent with the results obtained from TIMER, the GEPIA results indicated that HSF2 exhibited positive and significant correlations with gene markers of these immune cells in HCC (Table 2).

**TABLE 1:** Correlation analysis between HSF2 expression and gene markers of immune cells using TIMER.

Description	Gene markers	HCC			
		None		Purity	
		Cor	P	Cor	P
CD8^+^ T cell	CD8A	0.18	***	0.224	***
	CD8B	0.116	*	0.164	**
T cell (general)	CD3D	0.155	**	0.206	***
	CD3E	0.168	**	0.236	***
	CD2	0.153	**	0.22	***
B cell	CD19	0.249	***	0.275	***
	CD79A	0.195	***	0.25	***
Monocyte	CD86	0.301	***	0.386	***
	CSF1R	0.235	***	0.31	***
TAM	CCL2	0.142	**	0.187	***
	CD68	0.243	***	0.287	***
	IL10	0.301	***	0.361	***
M1	IRF5	0.426	***	0.431	***
	NOS2	0.147	**	0.151	**
	PTGS2	0.27	***	0.34	***
M2	CD163	0.213	***	0.263	***
	VSIG4	0.194	***	0.244	***
	MS4A4A	0.204	***	0.261	***
Neutrophils	CEACAM8	0.087	0.094	0.105	0.051
	ITGAM	0.319	***	0.368	***
	CCR7	0.175	***	0.226	***
Natural killer cell	KIR2DL1	0.022	0.671	0.026	0.634
	KIR2DL3	0.167	**	0.206	***
	KIR2DL4	0.145	**	0.17	**
	KIR3DL1	0.103	*	0.114	*
	KIR3DL2	0.141	**	0.168	**
	KIR3DL3	0.009	0.867	0.006	0.909
	KIR2DS4	0.1	0.054	0.119	*
Dendritic cell	HLA-DPB1	0.146	**	0.194	***
	HLA-DQB1	0.09	0.0841	0.133	*
	HLA-DRA	0.193	***	0.242	***
	HLA-DPA1	0.199	***	0.258	***
	CD1C	0.162	**	0.196	***
	NRP1	0.441	***	0.463	***
	ITGAX	0.34	***	0.413	***

**TABLE 2:** Correlation analysis between HSF2 expression and gene markers of immune cells using GEPIA.

Description	Gene markers	HCC	
		R	P
B cell	CD19	0.25	***
	CD79A	0.19	***
T cell (general)	CD3D	0.11	*
	CD3E	0.15	**
	CD2	0.13	*
CD8^+^ T cell	CD8A	0.17	**
	CD8B	0.11	*
Monocyte	CD86	0.3	***
	CSF1R	0.27	***
TAM	CCL2	0.13	*
	CD68	0.27	***
	IL10	0.29	***
M1	IRF5	0.42	***
	NOS2	0.19	***
	PTGS2	0.29	***
M2	CD163	0.097	0.062
	VSIG4	0.2	***
	MS4A4A	0.22	***
Neutrophils	CEACAM8	0.12	*
	ITGAM	0.38	***
	CCR7	0.17	**
Natural killer cell	KIR3DL3	0.04	0.44
	KIR2DL1	0.1	*
	KIR2DL3	0.18	***
	KIR2DL4	0.18	***
	KIR2DS4	0.071	0.18
	KIR3DL1	0.059	0.26
	KIR3DL2	0.22	***
Dendritic cell	HLA-DPA1	0.21	***
	HLA-DPB1	0.17	**
	HLA-DQB1	0.0022	0.97
	HLA-DRA	0.2	***
	CD1C	0.15	**
	NRP1	0.43	***
	ITGAX	0.36	***

Furthermore, HSF2 expression was also significantly correlated with the infiltration of subsets of T cells, including Th1, Th1-like, Th2, Treg, resting Treg cells, effective Treg cells, effective T cells, naïve T cells, effective memory T cells, resistant memory T cells, and exhausted T cells, according to the TIMER database (Table 3). In addition, the expression of many immune checkpoint genes, including CD274, CTLA4, HAVCR2, LAG3, PDCD1, PDCD1LG2 and TIGHT, was much higher in the HSF2 high-expression group than in the HSF2 low-expression group (Figure 8E). Interestingly, HSF2 expression was positively associated with multiple immune checkpoints, including PD-L1, PD-1 and CTLA-4 (Supplementary Figure S3A). The correlations between HSF2 and PD-L1, PD-1 and CTLA-4 expression were further confirmed in both the TIMER and GEPIA databases (Supplementary Figures S3B,C), revealing that increased expression of HSF2 was associated with immunosuppression in HCC. Furthermore, we investigated the effect of HSF2 expression on immune checkpoint blockade therapy in both TCGA and ICGC datasets and found that the HSF2 high-expression group exhibited a higher TIDE (tumor immune dysfunction and exclusion) score, which indicates a worse response to immunotherapy (Figure 8F).

**TABLE 3:** Correlation analysis between HSF2 expression and gene markers of different types of T cells using TIMER.

Description	Gene markers	HCC			
		None		Purity	
		Cor	P	Cor	P
Th1	TBX21	0.144	**	0.201	***
	STAT4	0.237	***	0.262	***
	STAT1	0.419	***	0.432	***
	IFNG	0.19	***	0.236	***
	TNF	0.28	***	0.344	***
Th2	GATA3	0.204	***	0.284	***
	STAT6	0.316	***	0.308	***
	STAT5A	0.426	***	0.457	***
	IL13	0.121	*	0.13	*
Tfh	BCL6	0.475	***	0.43	***
	IL21	0.113	*	0.14	**
Th17	STAT3	0.368	***	0.393	***
	IL17A	0.112	*	0.12	*
Treg	FOXP3	0.25	***	0.275	***
	CCR8	0.407	***	0.459	***
	STAT5B	0.546	***	0.551	***
	TGFB1	0.256	***	0.299	***
Effector T cell	CX3CR1	0.302	***	0.326	***
	FGFBP2	-0.036	0.493	-0.01	0.851
	FCGR3A	0.299	***	0.351	***
Naïve T cell	CCR7	0.175	***	0.226	***
	SELL	0.292	***	0.345	***
	TCF7	0.312	***	0.301	***
	LEF1	0.269	***	0.274	***
Effective memory T cell	PDCD1	0.256	***	0.296	***
	DUSP4	0.296	***	0.361	***
	GZMK	0.086	0.096	0.127	*
	IFNG	0.19	***	0.236	***
Resident memory T cell	CD69	0.258	***	0.321	***
	ITGAE	0.256	***	0.277	***
	CXCR6	0.178	***	0.244	***
Exhausted T cell	MYADM	0.533	***	0.556	***
	HAVCR2	0.308	***	0.399	***
	TIGIT	0.26	***	0.34	***
	LAG3	0.199	***	0.223	***
	PDCD1	0.256	***	0.296	***
	CXCL13	0.164	**	0.205	***
	LAYN	0.274	***	0.312	***
Resting Treg cell	FOXP3	0.25	***	0.275	***
	IL2RA	0.35	***	0.42	***
Effector Treg cell	FOXP3	0.25	***	0.275	***
	CTLA4	0.275	***	0.339	***
	CCR8	0.407	***	0.459	***
	TNFRSF9	0.421	***	0.477	***
Th1-like	CXCL13	0.164	**	0.205	***
	HAVCR2	0.308	***	0.399	***
	IFNG	0.19	***	0.236	***
	CXCR3	0.174	***	0.221	***
	BHLHE40	0.346	***	0.348	***
	CD4	0.273	***	0.311	***
General memory T cell	CCR7	0.175	***	0.226	***
	SELL	0.292	***	0.345	***
	IL7R	0.273	***	0.332	***
	CDH1	0.18	***	0.173	**
	CHD2	0.586	***	0.586	***
	VIM	0.293	***	0.355	***

### Prognostic Value of Heat Shock Factor 2 Expression Based on Immune Cell Infiltration in Patients With Hepatocellular Carcinoma

Because a high HSF2 expression level is associated with a poor prognosis and immune cell infiltration, we then investigated the relationship between HSF2 expression and the prognosis of patients with HCC in related immune cell subgroups using the Kaplan-Meier plotter database. Interestingly, patients with HCC presenting high HSF2 expression and either increased or decreased infiltration of B cells, CD4^+^ T cells, CD8^+^ T cells, macrophages, NK cells, Th1, Th2 and Treg cells had a shorter OS (Supplementary Figures S4A-H). Moreover, patients with HCC presenting high HSF2 expression and either increased or decreased infiltration of B cells, macrophages, NK cells, Th1 cells, Th2 cells and Treg cells had a shorter RFS (Supplementary Figures S5A-H).

### Silencing Heat Shock Factor 2 Prevented the Proliferation of Hepatocellular Carcinoma Cells

**FIGURE 9:**
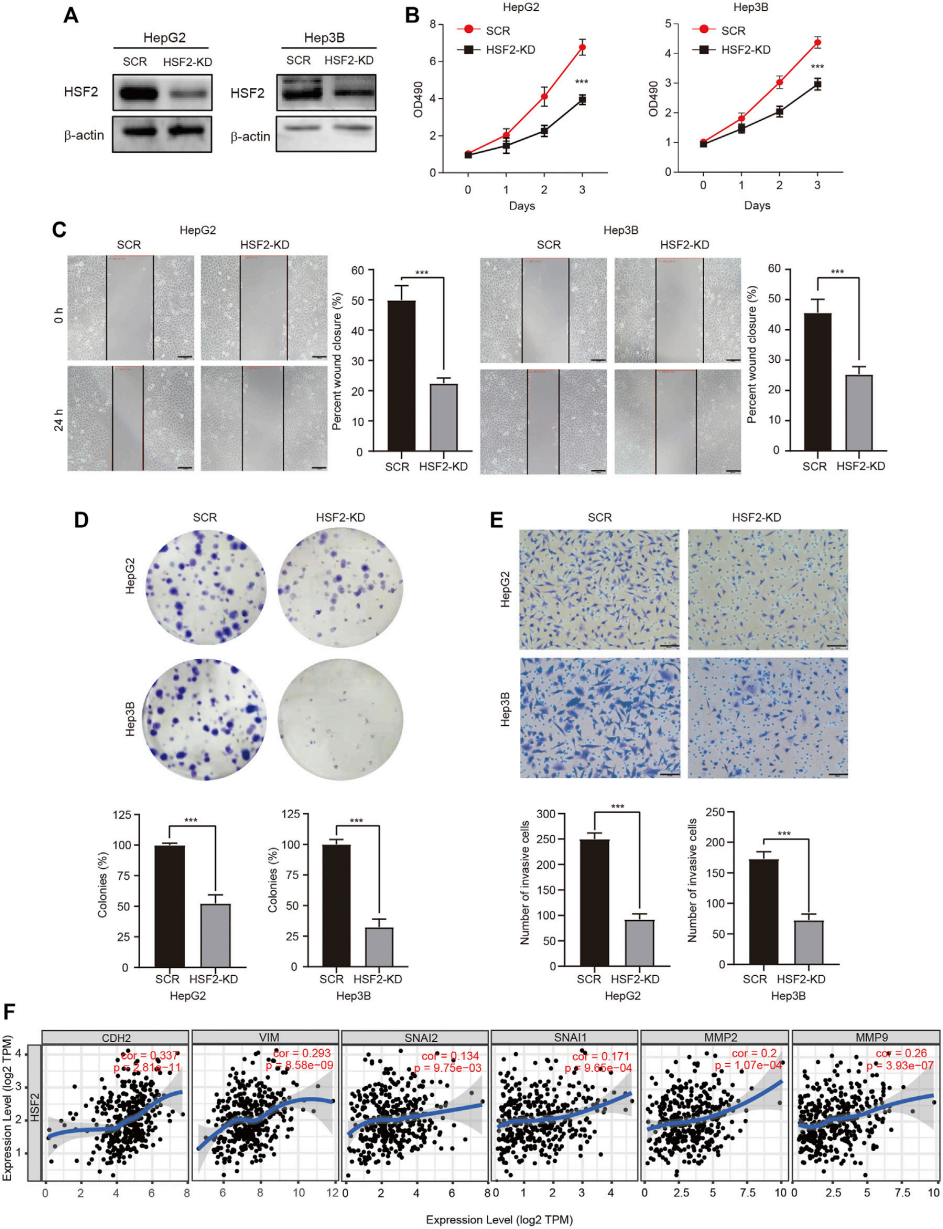
HSF2 mediates the proliferation and malignancy of HCC cells. **(A)** The knockdown efficacy was confirmed by western blotting. **(B)** Cell viability was examined using a CCK-8 assay. **(C)** The migration of HepG2 and Hep3B cells was detected using a wound healing assay. Scale bar, 100 μm. **(D)** Representative images of colonies formed by HepG2 and Hep3B cells. **(E)** The invasive ability of HepG2 and Hep3B cells with HSF2 knockdown was detected by performing Matrigel-coated Transwell assay. Numbers of invaded cells were counted. Scale bar, 100 μm. **(F)** Scatterplots showing the correlations of HSF2 expression and CDH2, VIM, SNAI1, SNAI2, MMP2 and MMP9 in HCC using the GEPIA2 database. Data are presented as the means ± SD. *p < 0.05, **p < 0.01, ***p < 0.01.

We genetically knocked down (KD) HSF2 in HepG2 and Hep3B cells to verify the oncogenic role of HSF2 in HCC. The knockdown efficacy was examined using western blotting (Figure 9A). The viability of HSF2-KD HepG2 and Hep3B cells was significantly decreased (Figure 9B). The migration and colony formation abilities of HepG2 and Hep3B cells were also dramatically inhibited when HSF2 was silenced (Figures 9C,D). Matrigel-coated Transwell assays revealed that HSF2 knockdown significantly reduced the invasion ability of both HepG2 and Hep3B cells (Figure 9E). In addition, the correlations between HSF2 expression and EMT-related and invasion-related markers were investigated using the TIEMR database. HSF2 expression was correlated with CDH2 (N-cadherin), VIM (vimentin), SNAI1 (Snail), SNAI2 (Slugc), MMP2 and MMP9 (Figure 9F). Taken together, HSF2 increased the viability, proliferation and metastasis of HCC cells.

## Discussion

Liver cancer is one of the most common and aggressive malignancies and ranks as the fourth leading cause of cancer-related mortality worldwide (Bray et al., 2018; Forner et al., 2018). In most high-risk HCC areas, including China, the predominant causes are chronic hepatitis B virus (HBV) infection, aflatoxin exposure and alcohol consumption (Fu and Wang, 2018). Although remarkable advances have been achieved in surgery and targeted therapeutic drugs, the prognosis is still unsatisfactory due to recurrence, metastasis and frequent drug resistance [1]. Therefore, improving the early diagnosis rate of HCC, discovering new biological markers for evaluating prognosis, and discovering new targets for antitumor therapy have become hot topics in HCC research. In the present study, we clearly showed that HSF2 expression in HCC is significantly upregulated based on bioinformatics analysis using the TIMER, HCCDB, GEO and TCGA databases (Figure 1). HSF2 expression closely correlates with multiple clinicopathological parameters, including age, sex, clinical stage, histological grade and metastasis, in patients with HCC (Figure 2). Subsequently, the clinical prognostic significance of HSF2 expression in patients with HCC was confirmed. Kaplan-Meier survival analyses indicated that patients with HCC presenting high HSF2 expression had a markedly worse survival rate than those with low HSF2 expression (Figure 3). A Cox hazard regression analysis was performed, and a nomogram model was constructed to investigate the prognostic value of HSF2 in HCC (Figure 4). According to the patient samples in the cBioPortal database, approximately 1.7% of patients with HCC exhibit genetic alterations in HSF2 (Figure 5). We also revealed that most of the alterations in HSF2 were deep depletion in HCC, and these alterations of HSF2 were correlated with shorter DFS and PFS. These results substantiated that HSF2 may be an independent prognostic biomarker for HCC.

Compared to normal cells, cancer cells are more dependent on HSF due to the need to strengthen chaperone induction to adapt to various stresses induced by changes in protein synthesis, misfolding, and subsequent overloading of the proteasomal degradation system (Akerfelt et al., 2010; Fujimoto and Nakai, 2010; Gomez-Pastor et al., 2018; Puustinen and Sistonen, 2020; Wang et al., 2020). HSF1 is substantially overexpressed in various types of cancer and regulates the noncanonical transcriptional program, which is critical for tumor development and progression (Mendillo et al., 2012; Shao et al., 2019; Zhang et al., 2021). In contrast to studies on HSF1, most studies on HSF2 have focused on protein misfolding diseases, aging, and the development of the embryo and sperm (Rallu et al., 1997; Shinkawa et al., 2011; Widlak and Vydra, 2017). Because HSF2 silencing changed the stability of p53 and its cooperation with HSF1, HSF2 likely affects tumorigenesis (Lecomte et al., 2010). Several previous studies have indicated that HSF2 expression is altered in several types of cancer, including breast cancer, ESCC, lung cancer and prostate cancer. High levels of HSF2 facilitate the proliferation and invasion of breast cancer cells. HSF2 cooperates with zinc finger E-box-binding homeobox 1 (ZEB1) to upregulate the miR-183/-96/-182 cluster (Li et al., 2014). The miR-183/-96/-182 cluster then promotes the migration and survival of breast cancer cells by inhibiting the expression of the tumor suppressor RAB21 (Li et al., 2014). In breast cancer cells, HSF2 regulates ALG3 enzyme expression to enhance cell proliferation and migration (Yang et al., 2018). Moreover, ALG3 knockdown reduces tumor growth and HSF2 expression levels, indicating a feedback loop between HSF2 and ALG3 (Yang et al., 2018). Moreover, miR-202 is aberrantly expressed in ESCC and negatively regulates apoptosis by directly targeting HSF2 and subsequently affecting HSP70 expression (Meng et al., 2017). HSF2 is aberrantly expressed in lung cancer and influences cancer cell growth and migration by acting as an upstream regulator of HSPs (Zhong et al., 2016). However, HSF2 expression is significantly reduced in prostate cancer tissues compared to normal controls, and the decrease in HSF2 expression is related to the metastasis of prostate cancer (Björk et al., 2016). Bioinformatics analysis of RNA-sequencing data suggested that HSF2 may be associated with the development of thyroid carcinoma by regulating SERPINA1 and FOSB expression (Lu and Zhang, 2016). The Wnt/β-catenin signaling pathway is known to participate in carcinogenesis, and its deregulation has been established in several cancers. HSF2 was identified as a novel target of Wnt/β-catenin signaling in HCC using a genome-wide comparative screening approach (Kavak et al., 2010). Recently, HSF2 was shown to interact with euchromatic histone lysine methyltransferase 2 (EHMT2) to downregulate fructose-bisphosphatase 1 (FBP1) (Yang et al., 2019). FBP1 silencing promoted HIF1α activation and the expression of glucose transporter 1 (GLUT1), hexokinase 2 (HK2), and lactate dehydrogenase A (LDHA), thereby enhancing aerobic glycolysis in HCC (Yang et al., 2019). These findings support the hypothesis that HSF2 is an upstream regulator of oncogenic mechanisms relevant to cancer progression and invasion, and it may become an attractive therapeutic target.

Immunotherapy has drastically advanced with the clinical success of immune checkpoint inhibitors in treating several cancer types. A previous study showed that HSF2 is upregulated in ulcerative colitis and increases the production of inflammatory cytokines (Miao et al., 2014). The chronic inflammatory response is also closely associated with the occurrence of HCC. Recent studies have indicated that HCC is an immunogenic tumor (Sia et al., 2017). Immunotherapy has broad prospects for improving the prognosis and reducing the mortality of patients with advanced HCC (Pinter et al., 2021). The TME is strongly correlated with every pivotal aspect of HCC tumorigenesis, including tumor occurrence, progression, metastasis, recurrence, resistance to therapy and immune cell invasion (Quail and Joyce, 2013). Therefore, an in-depth understanding of the TME is very important for revealing its underlying molecular mechanisms and providing new strategies to improve the efficacy of immunotherapy. Various immune cell subtypes are present in the TME, and different types of immune cells have different functions. Our study revealed connections between HSF2 expression and immune cell infiltration levels in HCC using the TIMER and CIBERSORT databases (Figure 8). Increased levels of infiltrating B cells, CD4^+^ T cells, neutrophils, macrophages and dendritic cells were observed in the HSF2 high-expression group compared with the HSF2 low-expression group (Figure 8D). In fact, tumor immunity is often inhibited in patients with HCC, especially in the liver microenvironment, which is susceptible to developing immune tolerance to reduce the effect of immunotherapy. The most notable immune-suppressive mechanisms are immune checkpoint pathways, including the CTLA-4 and PD-1/PD-L1 pathways, which dampen T cell activation through ligand-receptor interactions (Sia et al., 2017; Pinter et al., 2021). Notably, HSF2 expression was significantly associated with the expression of PD-1, PD-L1 and CTLA-4 in our study (Supplementary Figure S3). The expression of many immune checkpoint genes, including CD274, CTLA4, HAVCR2, LAG3, PDCD1, PDCD1LG2 and TIGHT, was much higher in the HSF2 high-expression group than in the HSF2 low-expression group (Figure 8E). These results indicate that HSF2 may directly or indirectly modulate the expression of these immune checkpoint genes through unknown mechanisms. In addition, T cell activity was negatively modulated by several types of resident cells in the TME, such as Tregs and exhausted T cells. It is now well substantiated that FOXP3-expressing Treg cells not only inhibit aberrant immune response against self-antigens but also suppress anti-tumor immune response (Tanaka and Sakaguchi, 2017). Accumulating studies have indicated that a large number of Treg cells infiltrate into multiple types of cancer, including HCC (Tanaka and Sakaguchi, 2017; 2019). More importantly, infiltration of Treg cells into cancer tissues is usually correlated with poor clinical prognosis. Depletion of Treg cells could augment antitumor immune responses in animal models. In fact, Treg cells regulate not only T and B cells but also DC cells, NK cells and macrophages through numerous humoral and cell-cell contact mechanisms (Nishikawa and Koyama, 2021). Various molecules are associated with Treg-mediated immune suppression mechanisms, such as inhibition of antigen-presenting cells through CTLA-4, secretion of inhibitory cytokines (TGF-β, IL-10 and IL-35), and expression of granzymes, LAG3 and GITR (Nishikawa, 2014). Among these mechanisms, suppression via CTLA-4 and IL-2 consumption via CD25 seems to play key roles. CTLA-4 is a highly potent co-inhibitory molecule that is constitutively expressed on Treg cells. We found that HSF2 expression positively correlated with the expression of Treg cell markers (FOXP3, CCR8, STAT5B and TGFB1), resting Treg cell markers (LAYN and FOXP3) and effective Treg cell markers (IL2RA, FOXP3, CTLA-4, CCR8 and TNFRSF9) in HCC, suggesting that HSF2 may have the potential to activate Treg cells (Table 3). Markers of T cell exhaustion, including MYADM, HAVCR2, TIGIT, LAG3, PDCD-1 and CXCL13, were significantly associated with HSF2 expression in HCC (Table 3). In addition, NK cells are cytotoxic lymphocytes of the innate immune system that are capable of killing infected or tumor cells (Liu S. et al., 2021). Unlike the events required for T cell activation, NK cell activation is controlled by NK receptor interactions with the target cell and is independent of antigen processing and presentation. When activated, NK cells release cytotoxic granules to directly lyse cancer cells. NK cells also produce many chemokines and cytokines, including interferon-γ (IFN-γ) and TNF-α, and are therefore crucial in regulating adaptive immune responses (Shimasaki et al., 2020; Myers and Miller, 2021). The field of NK cell-based cancer therapy has gained great attention and is now a major area of immunotherapy innovation. In the present study, we observed that HSF2 was negatively and significantly correlated with infiltration of NK cells. This is another explanation for the low efficacy of immunotherapy for HCC with high HSF2 expression. Together, these results indicate a potential role for HSF2 in immunosuppression and immune escape.

In summary, the expression patterns, prognostic value, genetic alterations, effects on immune cell infiltration, and protein-protein interaction networks of HSF2 in patients with HCC were investigated. This comprehensive bioinformatics analysis indicated that HSF2 may be a new prognostic biomarker for HCC. Moreover, HSF2, which is strongly correlated with immune cell infiltration and immune molecules, may represent a new target for studying the immune evasion of HCC cells and potentially serves as an immunotherapeutic target for HCC. However, the present study has several limitations. First, although we investigated the correlation between the expression of HSF2 and several important genes, the correlation coefficient was relatively low. More experiments are needed to validate the relationship between HSF2 and these genes. Second, protein post-translational modification (PTM), including phosphorylation, methylation, ubiquitination, acetylation, sumoylation, neddylation and glycosylation, increases the functional diversity of the proteome by modifying proteins with functional groups. The functions of PTMs in the pathogenesis of cancer and the prognostic biomarkers related to cancer have attracted the attention of the scientific community around the world. Here, we investigated the methylation level of HSF2 promoter in HCC; however, the potential correlations between PTMs of HSF2, such as ubiquitination, acetylation and sumoylation, and HCC are still unexplored. Third, although we analyzed the relationships among HSF2, immune cell infiltration and immune checkpoints, further verifications from both *in vitro* and *in vivo* experiments are required. Additionally, studies using other techniques, such as chromatin immunoprecipitation assay with sequencing (ChIP-seq) analysis and single-cell RNA sequencing analysis, are needed to comprehensively examine the HSF2-specific molecular mechanisms in oncogenesis.

## Data Availability

The original contributions presented in the study are included in the article/Supplementary Material, further inquiries can be directed to the corresponding author.
