# Supplementary figures and images for: Integrated Bioinformatics Analysis Identifies Heat Shock Factor 2 as a Prognostic Biomarker Associated With Immune Cell Infiltration in Hepatocellular Carcinoma
Source: Front Genet. 2021 Nov 30;12:668516. doi: 10.3389/fgene.2021.668516 (PMC8669829; doi:10.3389/fgene.2021.668516)

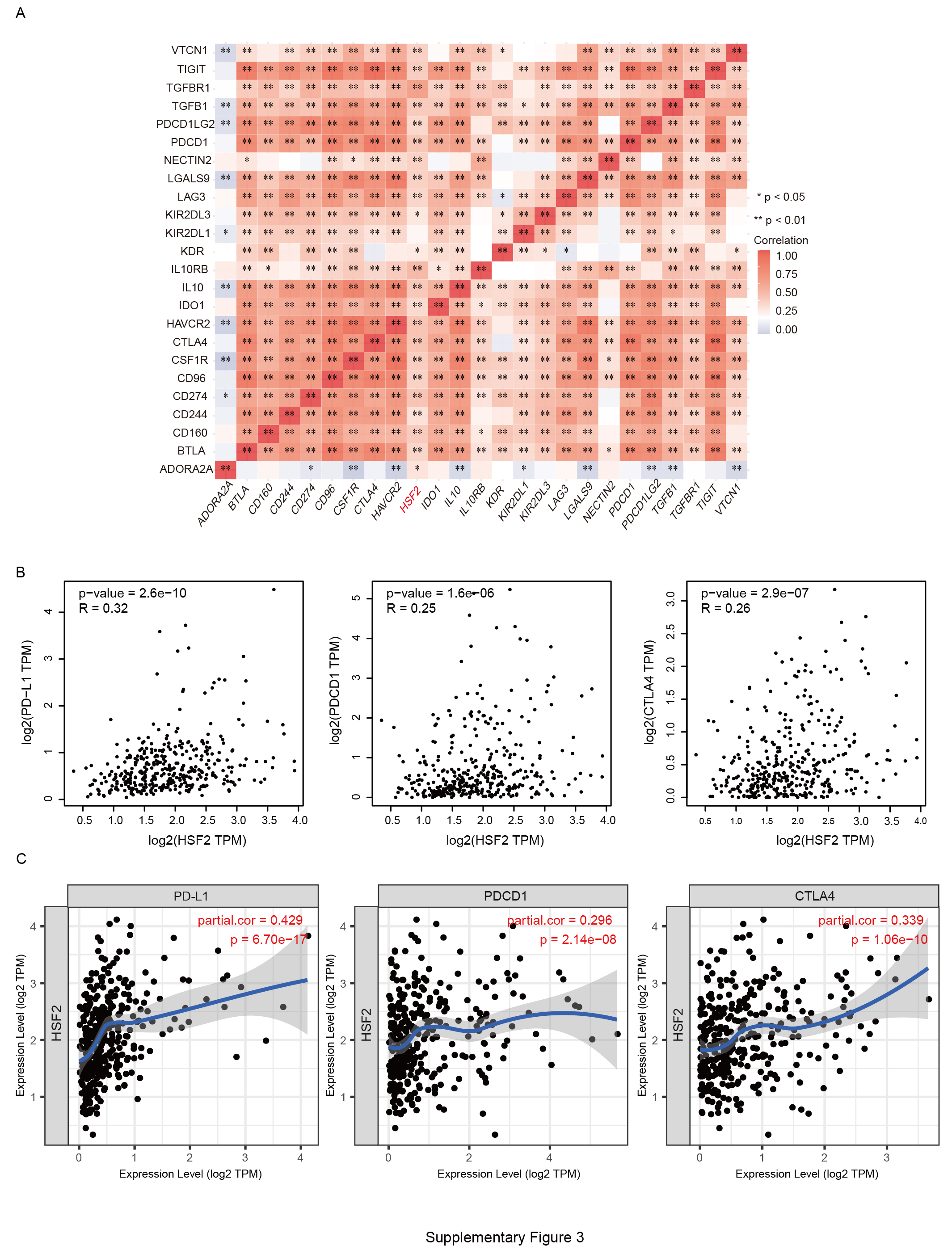

Supplement: Supplementary file 1 [file Image3.JPEG]

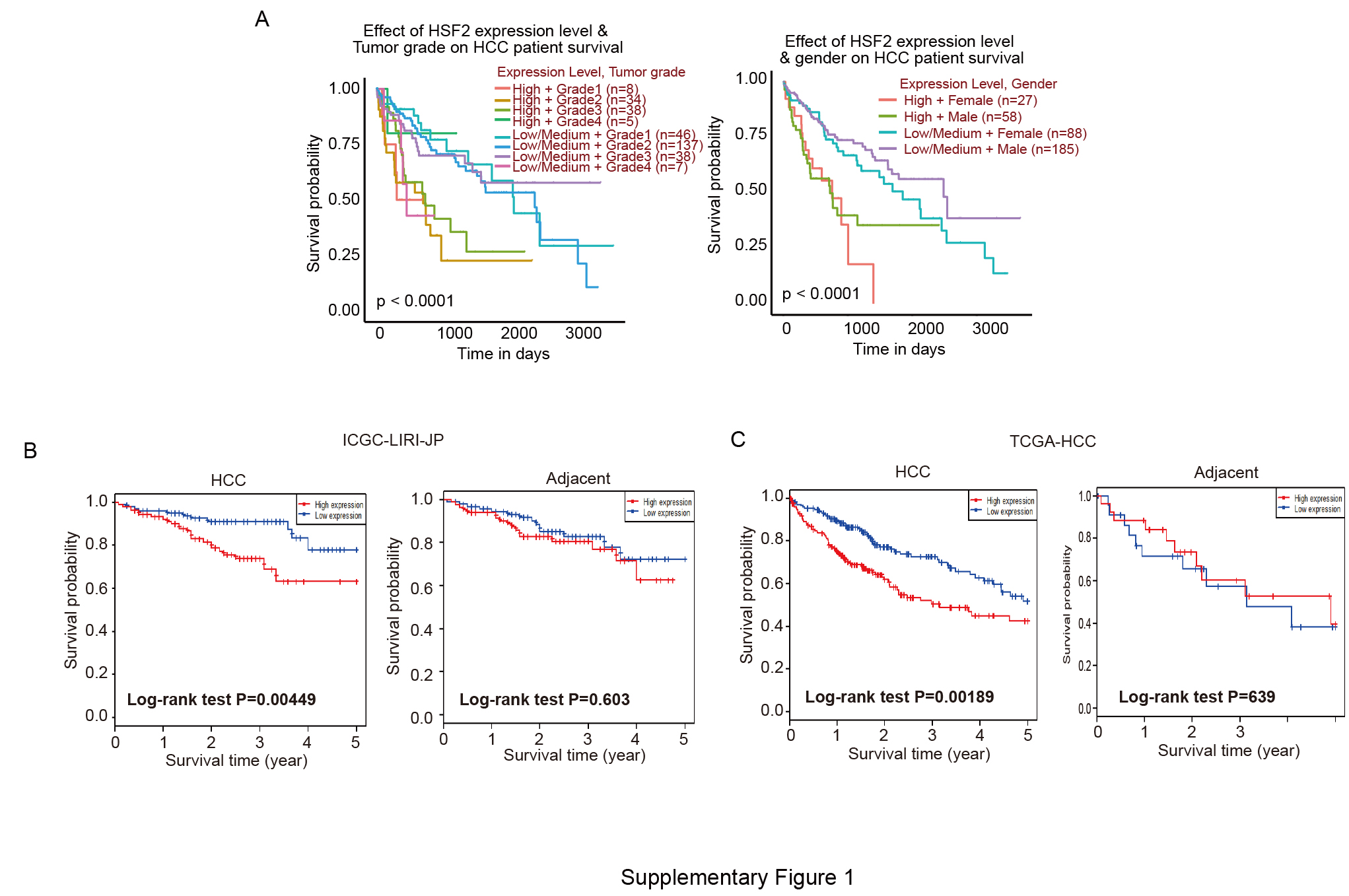

Supplement: Supplementary file 2 [file Image1.JPEG]

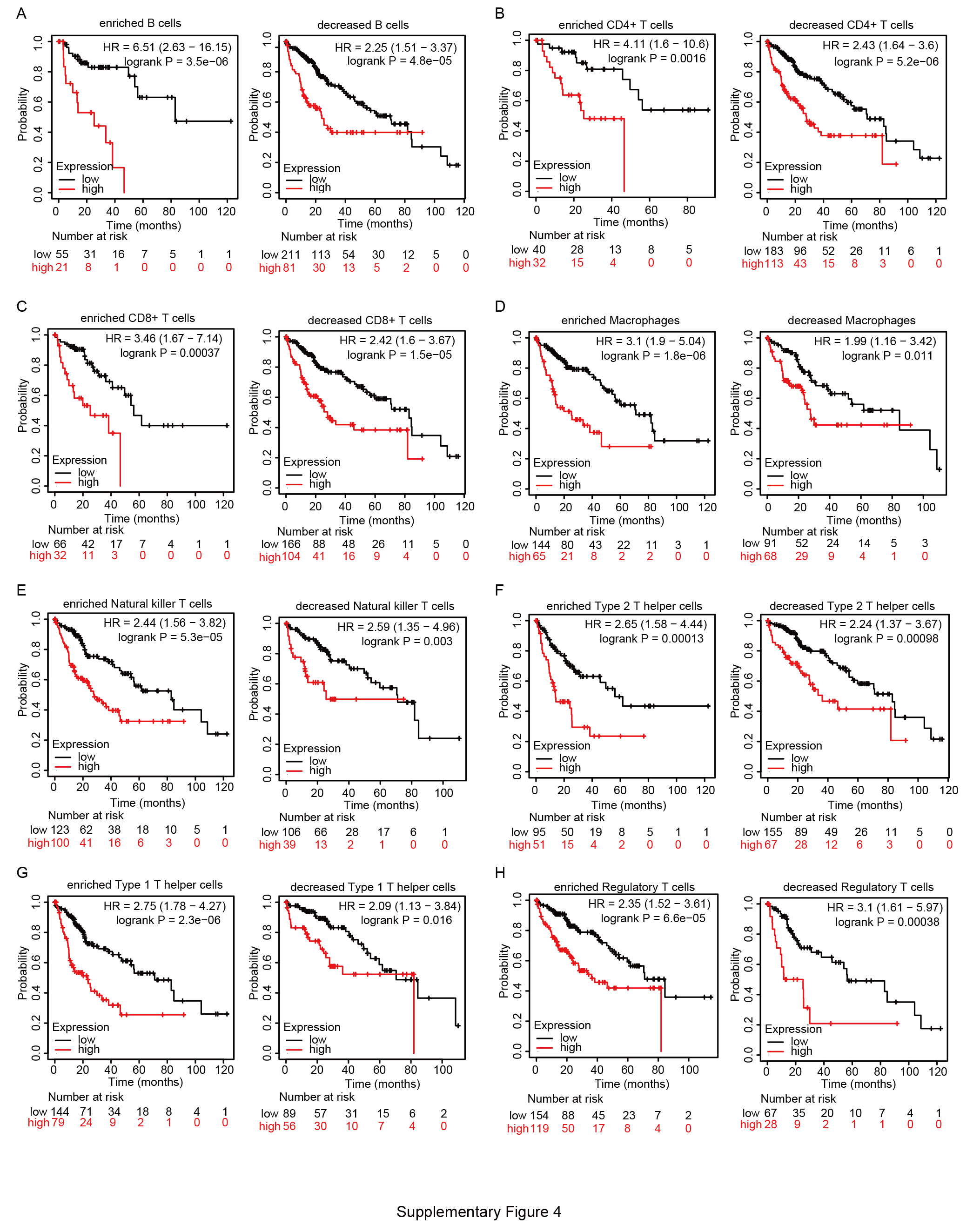

Supplement: Supplementary file 3 [file Image4.JPEG]

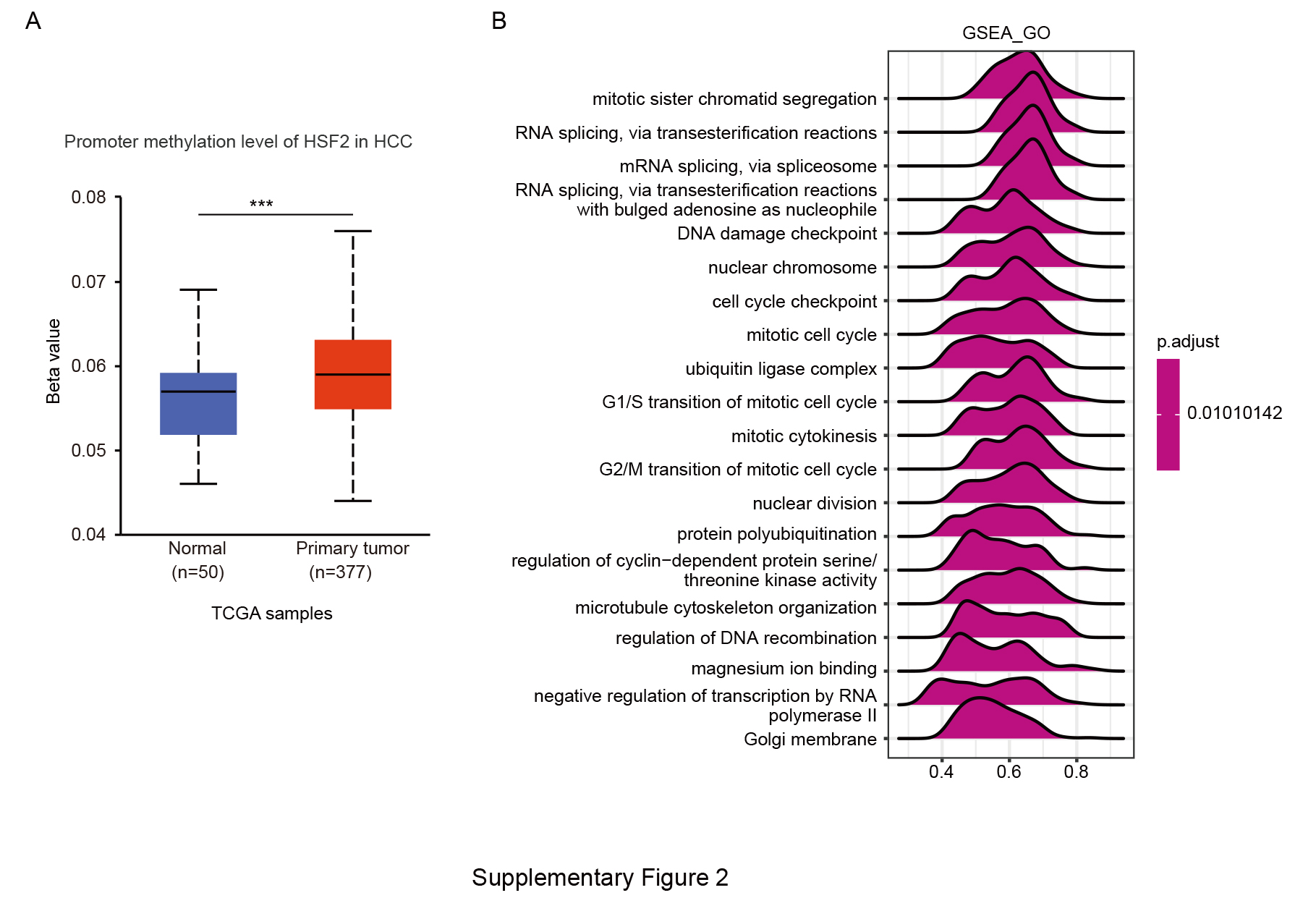

Supplement: Supplementary file 4 [file Image2.JPEG]

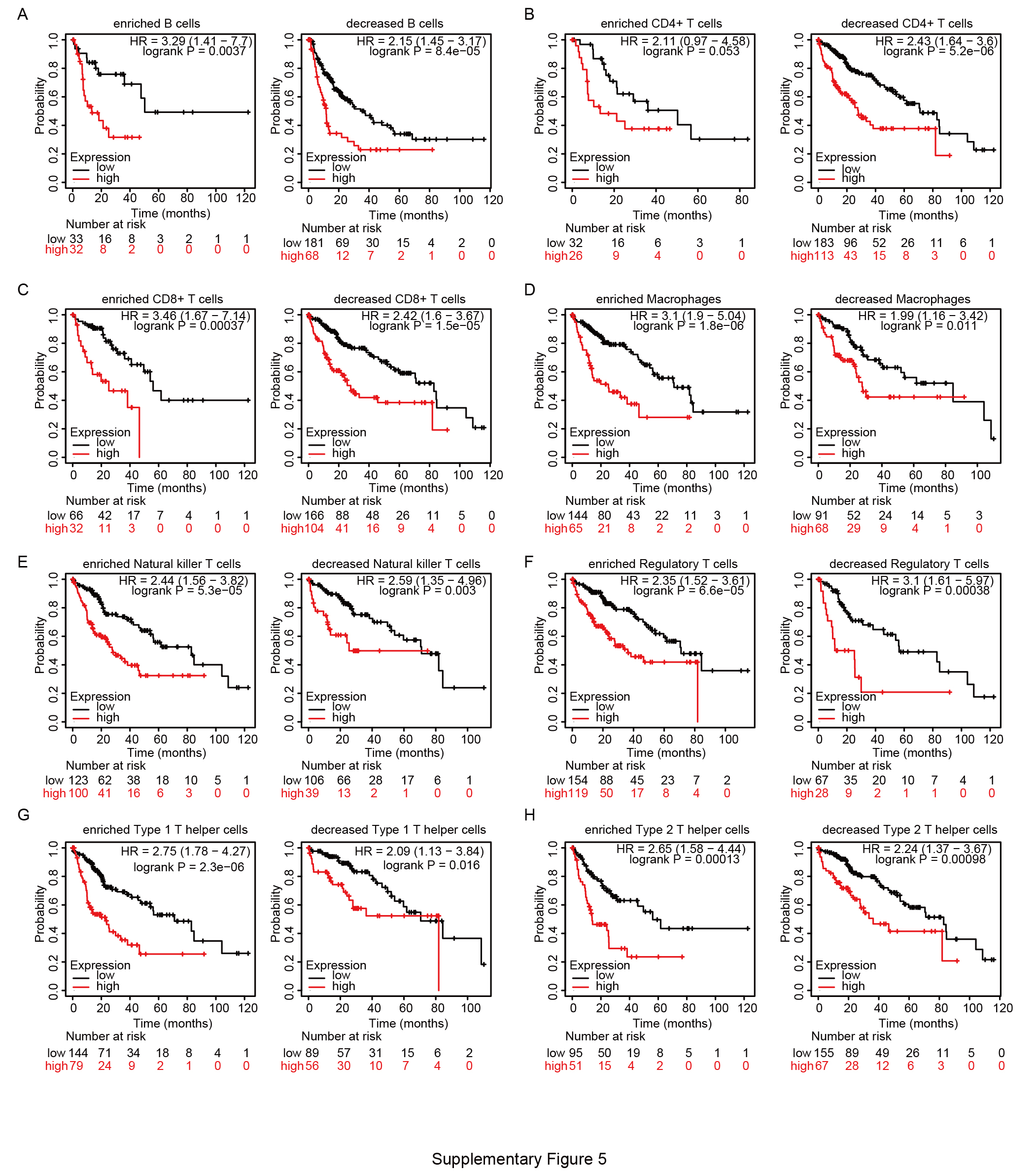

Supplement: Supplementary file 5 [file Image5.JPEG]
